# A new era of long-read sequencing for cancer genomics

**DOI:** 10.1038/s10038-019-0658-5

**Published:** 2019-09-02

**Authors:** Yoshitaka Sakamoto, Sarun Sereewattanawoot, Ayako Suzuki

**Affiliations:** Department of Computational Biology and Medical Sciences, Graduate School of Frontier Sciences, The University of Tokyo, 5-1-5 Kashiwanoha, Kashiwa, Chiba 277-8561 Japan

**Keywords:** DNA sequencing, Cancer

## Abstract

Cancer is a disease largely caused by genomic aberrations. Utilizing many rapidly emerging sequencing technologies, researchers have studied cancer genomes to understand the molecular statuses of cancer cells and to reveal their vulnerabilities, such as driver mutations or gene expression. Long-read technologies enable us to identify and characterize novel types of cancerous mutations, including complicated structural variants in haplotype resolution. In this review, we introduce three representative platforms for long-read sequencing and research trends of cancer genomics with long-read data. Further, we describe that aberrant transcriptome and epigenome statuses, namely, fusion transcripts, as well as aberrant transcript isoforms and the phase information of DNA methylation, are able to be elucidated by long-read sequencers. Long-read sequencing may shed light on novel types of aberrations in cancer genomics that are being missed by conventional short-read sequencing analyses.

## Introduction

Cancer cells harbor mutations in their genomes, parts of which affect the function of driver and tumor suppressor genes, resulting in the abnormal proliferation and initiation or progression of carcinogenesis. Drugs targeted at driver events show appreciable efficacy for shrinking tumor sizes. For example, *EGFR* tyrosine kinase inhibitors are effective for lung adenocarcinomas with *EGFR* mutations [1]. The identification of driver genes and the vulnerabilities of cancer cells have been energetically progressing by means of sequencing technologies.

Modern sequencing technologies are rapidly being developed to enable us to identify and characterize mutations in each cancer case more easily. Many consortiums, such as ICGC [2] and TCGA [3], have sequenced, analyzed, and reported on the genomic statuses specific to each cancer subtype. They have mainly focused on point mutations, such as single-nucleotide variants (SNVs) and short indels, because short-read sequencing techniques are generally being used for genotyping. However, other types of genomic aberrations are highly complicated. The detection and precise identification of various sizes of structural variants (SVs) and mutations in repetitive regions are challenging for short reads that are only a few hundred bases at the longest. The detection accuracy and precision are still limited, even though many bioinformatics tools and pipelines have been developed for these tasks (e.g., Pindel, DELLY2, Manta, SvABA) [4–7]. Short reads also lack the phasing information of each allele, which means that we are missing out on which alleles the mutations occurred in. To complement the vulnerability of short-read sequencing, new sequencing technologies for longer DNA chains are highly desirable in the field of cancer genomics.

Many long-read sequencing technologies have been developed and utilized in recent years. For example, single-molecule real-time sequencing (SMRT) [8] is one of the long-read methods developed by Pacific Biosciences (PacBio). This method is based on a single-DNA polymerase attached in a zero-mode waveguide (ZMW), which is a nanostructure for fluorescence detection. Using SMRT sequencing, we can obtain long-read data longer than 10 kb. In a recent report, approximately half (at least 26%) of the reads were sequenced with ≥ 10 kb length, and these datasets were used for the construction of comprehensive catalogs of common SVs in the human genome [9].

Nanopore-type sequencers have been commercialized by Oxford Nanopore Technologies. Protein nanopores are arrayed on a membrane to detect changes in an electrical current when a DNA or an RNA molecule passes through the pore, permitting direct sequencing of the molecules. MinION is a portable long-read sequencing platform with low initial costs capable of obtaining >5 Gb in each run. The library preparation is also simple to conduct and takes only ~48 h for each sequencing. Furthermore, a larger platform, PromethION, can achieve ~10 times the sequencing output of MinION. In our study, we used both MinION and PromethION for whole-genome sequencing of the lung cancer cell line LC2/ad. The lengths of the mapped reads are ~16 and 14 kb on average, respectively (up to 32 kb) [10]. For much longer reads, Jain et al. [11] reported a protocol for generating ultra-long reads (up to > 800 kb) to sequence and assemble the human genome with the intention of characterizing the difficult regions that include repetitive sequences and complicated structural variations. Correspondingly, it is also reported that these long reads could be used to probe into regions that were previously inaccessible by conventional short-read sequencers [12], underlining the advantages that long-read sequencing could offer. Oxford nanopore sequencers enable us to easily obtain long reads although they suffer a relatively lower sequencing accuracy than that from short-read sequencing technologies.

In contrast to these physical long-read sequencers, researchers can also obtain synthetic long-read sequences reconstructed from short-read sequencing with barcode sequences attached to each high-molecular-weight DNA molecule. 10x Genomics released a linked-read technology based on the generation of oil-droplet-containing barcoded gel beads, reaction reagents, and DNA molecules ( > 100 kb) using the Chromium system. Only 1 ng of genomic DNA is needed. This method provides the phase information of SNPs for haplotyping the genome (N50 phase block lengths ranged from 0.9–2.8 Mb) [13] and enables the detection of SVs by following the molecular barcodes specific to each large DNA fragment.

Long-read sequencing is now becoming more prevalent, and thus, cancer studies using long-read information have been rapidly increasing and continuously progressing in order to decipher complicated cancer genomes. Here, we introduce recent long-read analyses for cancer research and new perspectives of cancer genomics brought by long-read sequencing (Table 1).

**Table 1:** Recent research of cancer genomics using long-read sequencing technologies

Category	Sequencing technology	Cancer	References	Published year
Phasing	ONT	Lung cancer	[16]	2017
	ONT/linked read	Lung cancer	[21]	2018
Structural variation	ONT	Brain tumors	[15]	2017
	Linked read	Gastric cancer	[33]	2017
	Linked read	Prostate cancer	[34]	2018
	PacBio	Breast cancer	[35]	2018
	ONT	Lung cancer	[42]	2019
	ONT	Lung cancer	[10]	2019 (preprint)

## Cancer genome sequencing with long reads

The strength of long-read sequencing is that it is suited for elucidating allele-resolution mutation statuses and the complete structures of complicated cancer genomes. While the representative physical long-read platforms such as PacBio and Oxford Nanopore sequencers produce sequences with lower base qualities than those of short-read sequencing platforms such as Illumina sequencers, this shortcoming could be circumvented when genotyping large genome aberrations, such as copy-number variants (CNVs) and SVs. This approach has been taken in various diseases [14], including cancers [15–18]. Moreover, with a careful application of either the long reads alone or in tandem with a more accurate conventional short-read sequencing, single-base-level resolution aberrations such as SNVs and short indels could be genotyped. For example, SNVs and short indels in cancer-related genes such as *EGFR*, *KRAS*, *NRAS*, and *NF1* could be detected using only MinION reads by considering reads without errors in ± 3 bases around the mutation [16]. Also in the same study, variant detection limits were investigated using serially diluted samples (1 to 50% of mutant cells mixed with wild-type cells). The variants could be detected at the expected ratio although it is difficult to detect mutations with low-variant allele frequencies (<10%) because high rates of sequencing and mapping errors in nanopore sequences [16, 19]. In addition, several studies resort to hybrid methods utilizing short-read data to correct the errors in long reads [20].

**Fig. 1:**
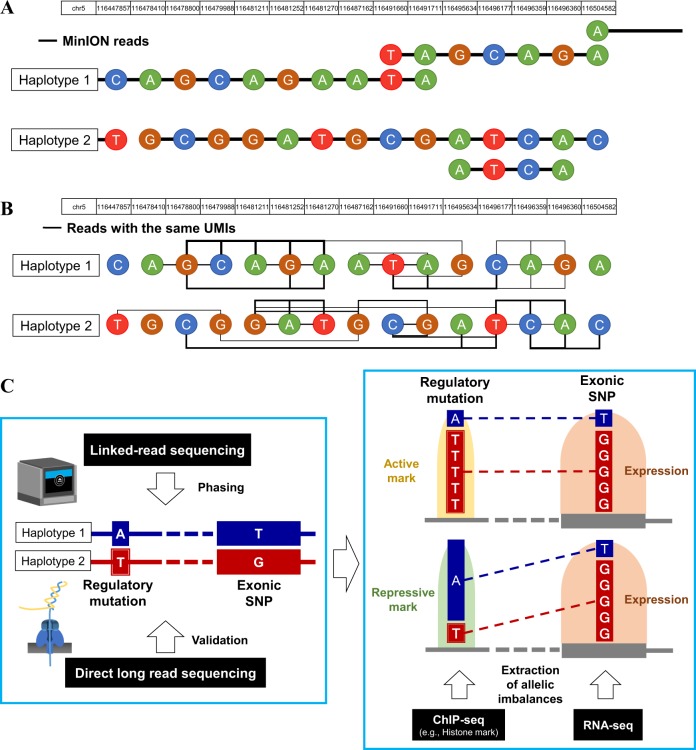
Phasing of the allelic relationships between noncoding regulatory regions and exonic regions. **a**, **b** An example of phasing by both physical (**a**) and synthetic (**b**) long-read technologies. The lines show an allelic relationship of multiple single- nucleotide variants (SNVs) of the *SEMA6A* gene in the H1975 cell line (previously reported in Sereewattanawoot et al. [21]). Each SNV is represented in a circle. **c** The scheme for identification of regulatory mutations affecting transcription and gene expression by phasing analysis [21]. Heterozygous regulatory mutations are associated with exonic variations in allele resolution using linked-read sequencing (left). The phasing results are validated by physical long reads. Allelic transcription and expression are also considered to evaluate the impact on regulatory mutations (right). The RNA-seq and ChIP-seq data are used to measure the transcription and expression statuses in allele level

One of the advantages of long-read sequencing is phasing genomic mutations in single-allele resolution (Fig. 1). For example, *EGFR* primary and secondary mutations (L858R and T790M, respectively) in the H1975 lung adenocarcinoma cell line were phased by both physical and synthetic long-read sequencing [16, 21]. In our MinION sequences, we found that both L858R and T790M mutations were in the same allele (72% of the transcript reads) and the other allele was a wild type (22% of the reads; the remaining 6% of the reads included sequencing errors or minor allele fractions) [16]. More than half of lung adenocarcinoma patients in Japan harbor *EGFR* mutations [22], and resistance to EGFR tyrosine kinase inhibitors (TKIs) and relapse are associated with tumor clones harboring secondary-resistant mutations, which become more common during and after treatment. A large fraction of patients with TKI resistance have secondary- or tertiary-resistant mutations in *EGFR* itself [23, 24]. Previous studies reported that the combination of those mutations and their allelic mutual relationships in each copy of the genome are associated with the sensitivity of each EGFR TKI [25]. Owing to the emphasis on the influences of combinations and the allelic compositions that mutations have in understanding the mechanisms of cancer evolution and survival while undergoing treatment with molecular targeted drugs, long-read sequencings could become the new standard for both genotyping genes for the development of anti-cancer drugs and tailoring the right treatment for each individual.

Further, phasing is vital in understanding the functional relevance of noncoding mutations. In cancer cells, there are numerous mutations in promoters and enhancers, parts of which affect aberrant transcription and consequently the abundance of gene expression. For example, mutations in the *TERT* promoter region frequently occur in diverse types of cancers, and mutations in these hotspots create de novo-binding sites of oncogenic transcription factors in ETS family genes [26–28]. In cancer cell lines, only the mutant *TERT* alleles are expressed, which indicates that the promoter mutation created a de novo transcription factor binding site and activated transcription and expression in the mutant allele [29]. Using short-read sequencing data, direct associations between the promoter and the downstream exonic region at the allele level could not be realized because those loci are far more than hundreds of bases apart and cannot be covered by a single or a small number of short reads. In a previous study by our group, we attempted to associate regulatory mutations with exonic variants in lung cancer cell lines using linked-read technology [21, 30] (left panel, Fig. 1c). By using a whole-exome plus regulome bait, on average, cumulatively 387 Mb of genomic regions were phased, and parts of the phasing results were validated by MinION physical long reads. Both the linked-read and physical long-read data are provided in the database DBKERO (http://kero.hgc.jp/) [30]. We additionally analyzed the ChIP-seq of histone modifications and RNA-seq [31] to evaluate which allele was transcriptionally activated/inactivated and consequently expressed in the cancer cells (right panel, Fig. 1c). As a result, >100 regulatory mutations in 23 lung-cancer cell lines were characterized as candidates that might affect transcription and gene expression [21]. As an example, we showed that a regulatory mutation in the *NFATC1* gene could be observed in RERF-LC-Ad1 with an addition of long-read allele phasing, and this regulatory SNV created a de novo-binding site of the ETS transcription factors that affect the allele-specific activation of *NFATC1* expression [21].

**Fig. 2:**
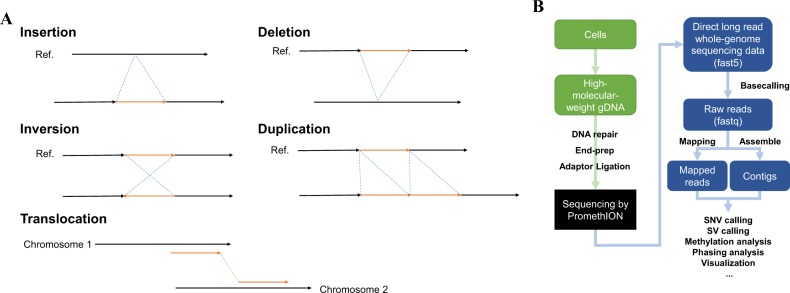
Structural variants in lung cancers identified by PromethION. **a** Five representative types of structural variants in cancer genomes. Ref.: Reference sequences. **b** The workflow of direct, long-read whole-genome sequencing analysis by PromethION [10]. Intact high-molecular-weight genomic DNA is extracted from fresh or frozen cells. For the construction of sequencing libraries of PromethION, DNA repair, end-prep, and adaptor ligations are conducted according to the manufacturer’s protocols. Sequencing starts after the library is loaded to the flow cell. Base calling is performed to obtain the sequencing read data, because the data are first provided in the fast5 file format. After getting the fastq files, the reads are mapped to the reference genome and/or assembled, and various analyses are conducted, such as SNV/SV calling, methylated base calling, phasing analysis, and visualization by genome viewers

Long reads are also utilized in the detection of SVs. SVs are defined as large aberrations >1 kb [32], such as large indels, inversions, and duplications, or chromosomal rearrangements, such as translocations (Fig. 2). To detect these aberrations, long reads were first mapped to a human reference genome, and by utilizing split-reads, which are reads that are composed of parts that could be uniquely mapped to different regions of the genome, the distinct sequences of the genomic regions evidenced by those reads were then employed to detect breakpoints of SVs. Short reads are also able to be used in this manner, but large and complex SVs and repetitive regions would be impossible for them. Norris et al. [17] reported that they attempted to use MinION to detect SVs and successfully identified cancer-related SVs in the *CDKN2A* and *SMAD4* tumor suppressor genes. In a report by a different group, they focused on the mutation status of *FGFR2* in gastric cancer metastases using linked-read sequencing. They found and experimentally validated structural rearrangements of *FGFR2* as the driving factors in metastatsis [33]. For prostate cancers, a research group reported a linked-read sequencing analysis of genomes in castration-resistant patients [34]. They associated a tandem duplication phenotype with *CDK12* inactivation and identified an *AR* enhancer duplication in most of the patients. Nattestad et al. [35] demonstrated the detection of SVs in genomes of a breast cancer cell line utilizing both long-read sequencing and short-read sequencing. Interestingly, they found that *ERBB2* amplification appeared within complex rearrangements at chromosome 8, which could only be precisely identified by long-read sequencing. Furthermore, in our recent study, we performed whole-genome long-read sequencing of lung cancer cell lines and clinical samples using PromethION [10]. We obtained 47 Gb per sample on average and mapped the long reads to the human reference genome using the minimap2 [36] software package. We were able to detect profoundly complicated SVs with combinations of local duplication, inversion and/or deletions in tumor suppressor genes. The functions of these genes that harbor the SVs were lost at the transcript and protein levels. We could also detect these complicated SVs in clinical samples in the same manner. Although these complicated SVs could also be identified from short-read sequencing data by using various bioinformatics tools (e.g., GenomonSV [37]) aimed at detecting soft-clipped reads that are split into two different loci, the rate of false positives using that approach is relatively high, and their structures cannot be completely elucidated, complicating the evaluation of such results.

Long reads have enabled us to characterize aberrant genomic statuses that had been unclear by using conventional short-read sequencing analysis. However, the amplification of DNA libraries, which is a crucial step in detecting mutations of low frequencies, has become a much more formidable task, as PCR and hybridization limit the size of the fragments up to only a few kb. To remedy this, Cas9-Assisted Targeting of CHromosome segments (CATCH), a method based on the CRISPR-Cas9 system to isolate large genomic fragments, was developed [38]. Gabrieli et al. [39] reported that a large fragment of up to 200 kb, including the 80 kb *BRCA1* region, one of the breast and ovarian cancer-related genes, was obtained from PBMC using CATCH and sequenced by a MinION sequencer. They succeeded at sequencing the *BRCA1* region at ~70 × coverage per flow cell.

## Multi-layered analysis: transcriptome and epigenome

### Full-length transcriptome of cancer cells

Transcriptome analysis is also benefited by an application of long-read sequencing techniques. Long reads are able to completely cover full-length transcript sequences, and thus, structures of transcript isoforms can be determined by sequencing full-length complementary DNAs (cDNAs). In particular, fusion transcripts are known to be major driver events for carcinogenesis in several types of cancers, such as lung adenocarcinoma, which can be detected by long-read sequencing. Further, transcripts with aberrant structures are extremely likely to produce tumor unique neoantigens that are recognizable by immune cells; thus, they are an ideal marker for the selection of immune checkpoint inhibitors. For example, head and neck cancers harboring fusion transcripts produce fusion-derived neoantigens and respond to immunotherapy treatments even though the mutation burden is low and only a fraction of the immune cells infiltrate into the tumor tissues [40]. The investigators also verified that the fusion-derived neoantigens stimulated T-cell responses, emphasizing the importance of sequencing full-length transcripts and elucidating their complete structures.

PacBio sequencers could be applied for full-length cDNA sequencing (called Iso-seq) to detect splicing isoforms and fusion transcripts. This was demonstrated in SK-BR-3, the most studied cancer cell line as a model of breast cancer. Comprehensive genome and transcriptome sequencing were performed using PacBio SMRT sequencing to characterize the fusion transcripts in addition to genomic aberrations, including copy-number amplification and SVs [35].

A full-length transcriptome is also possible with Oxford Nanopore sequencers. Using an RNA spike-in mix, Oikonomopoulos et al. [41] determined that MinION could sequence full-length cDNAs and that the expression abundance showed a high correlation with other platforms (Illumina and PacBio). In a recent report by our group using MinION, we sequenced full-length transcripts on lung-cancer cell lines and showed that fusion transcripts, including *CCDC6-RET*, a driver mutation of the LC2/ad cell line, are promptly detected. Multiple heterozygous mutations, including SNVs associated with the sensitivity to molecular targeted drugs (e.g., *EGFR* mutations), could be sequenced and phased [42].

MinION, as a direct sequencing platform, is able to detect base modifications in RNA [43, 44], such as N6-methyladenine (m6A). Several studies reported that the molecular statuses of their writers and readers and consequent m6A patterns were implicated in the survival and maintenance of several cancer types, such as myeloid leukemia [45] and lung cancers [46], which indicates that modifications in full-length RNAs need to be profiled for identification of unknown characteristics and novel therapeutic targets in cancer cells. Further, the application of full-length cDNA sequencing at a single-cell level is now underway, allowing allelic and isoform-level information for individual cells on the transcriptome layer [47].

### Cancer epigenomics in long-read sequencing

DNA modifications play an important role in various biological events through transcriptional regulation. In cancer cells, we often observe genome-wide hypomethylation causing chromosomal instability [48]. Furthermore, hypermethylation specifically occurs in CpG islands in promoters of tumor suppressor genes, resulting in the silencing of genes such as cell cycle regulators and mismatch repair factors [49, 50]. Bisulfite sequencing is one of the standard methods for profiling the DNA methylation status by converting unmethylated cytosine to uracil to distinguish between methylated and unmethylated cytosine. However, bisulfite-treated DNA is damaged and fragmented and is thus not suitable for long-read analysis. To address this, Yang et al. [51], in 2015, reported on the development of a long-read bisulfite protocol using a PacBio sequencer and profiled methylations of hematologically malignant cell lines in single contiguous molecules.

Favorably, Oxford nanopore sequencers can directly detect methylated DNA [52–54]. The ionic current between methylated and unmethylated DNAs could be distinguished using several computational methods, such as Tombo [55], nanopolish [53], and signalAlign [52]. Further, the current Oxford nanopore basecaller, Flappie, is capable of recognizing 5mC methylation in CpG sites during flip-flop base calling (https://github.com/nanoporetech/flappie), enabling us to easily profile epigenomic conditions by means of base modifications at the same time as genome sequencing.

## The challenge of long-read sequencing for cancer genomics

Complementing cancer genome sequencing by short-read sequencers, long-read analysis enables even more comprehensive information of cancer genomes to be collected, including complicated genomic aberrations, transcript isoforms, epigenomic base modifications, and their phase statuses. However, long-read sequencing technologies still have several barriers withholding their application in clinical sequencing settings. First, sequencing accuracy is now ~90% in physical long-read platforms, which is insufficient and complicates the precise detection of point mutations. Second, it is not always possible to obtain large enough intact samples of high-molecular-weight DNA and full-length RNA from clinical samples. Surgical specimens and biopsies are commonly preserved as formalin-fixed paraffin-embedded (FFPE) tissues for histopathological staining and long-term storage. DNA/RNAs from FFPE samples are highly fragmented and damaged, and so biobanking fresh frozen tissues is required. Further, high-yield library preparation and sequencing protocols for small amounts of samples are still lacking in physical long-read techniques. While whole-genome/transcriptome amplification methods can be used increase the DNA/RNA amounts, direct sequencing of the original molecules is preferred to avoid the size limitations of the sample fragments caused by amplification and to pinpoint any base modifications in the molecules.

The development of analytical methods that could take full advantage of long-read sequencing has become one of the most important issues in bioinformatics. We now have various tools for base calling [56–58], genome assembly [59, 60], base polishing [61, 62], mapping [36, 63, 64], and phasing using long-read data. Especially for detecting mutations, various types of genomic aberrations, including SVs, need to be precisely detected. There are many existing tools that could call SVs [65–67] from long reads. However, none are resilient enough to rectify the higher sequencing error rates in physical long reads. Furthermore, we strongly desire visualization methods for complicated cancer genome structures deciphered by long-read sequencing. Graph-based representations have recently been accelerated in order to complement the linear-based methods to visualize and study genetic variations [68]. Although at present, there are already a number of methods for analyzing and visualizing genomes based on graph structures [69–73], graph genomes should be used more widely for cancer genome analysis.

By sequencing with current long-read technologies, we found that genes such as *MYC* [10] have exceptionally complicated genomic aberrations in the cancer genome. The regions around those genes are frequently rearranged and amplified on a megabase scale. Even current “long reads” cannot elucidate such regions because the reads are only tens or hundreds of kb in length. In such cases, the simultaneous use of optical mapping methods (e.g., Bionano Saphyr) that are specifically designed to visualize large-scale alterations could alleviate the issues. Further, in human genomes, there are numerous highly abundant repetitive regions, including centromeres and telomeres, and there are also ambiguous sequences in the reference genome. Details of mutation statuses in said regions and their functional relevance are still unknown. Further development and promotion of long-read sequencing are needed to thoroughly resolve cancer genomes and decode these genomic regions.
